# Population pharmacokinetic modeling and simulation-informed ceftazidime dosing in Chinese neonates using quantitative dried blood spot micro-sampling

**DOI:** 10.1128/aac.01810-25

**Published:** 2026-04-20

**Authors:** Qiufen Yin, Yao Chen, Jianmei Lv, Zhi Zheng, Xiaoyan Zhao, Huayan Chen, Feifan Xie, Hanxi Yi, Quanyao Chen

**Affiliations:** 1 Division of Biopharmaceutics and Pharmacokinetics, Xiangya School of Pharmaceutical Sciences, Central South University 506618 , Changsha, China; 2 Clinical Trial Institution, Scientific Research and lnnovation Center, Women and Children’s Hospital, School of Medicine, Xiamen University 205357, Xiamen, China; 3 Department of Neonatology, Women and Children’s Hospital, School of Medicine, Xiamen University 205357, Xiamen, China; 4 Department of Pathology, School of Basic Medical Science, Central South University 614453 , Changsha, China; University of Houston, Houston, Texas, USA

**Keywords:** ceftazidime, population pharmacokinetics, neonates, postmenstrual age

## Abstract

Ceftazidime is widely used off-label to treat neonatal infections, underscoring the need for optimized dosing strategies. However, pharmacokinetic (PK) studies in neonates are limited by the challenges of conventional blood sampling. This study applied dried blood spot (DBS) micro-sampling to develop a population PK (PopPK) model to support appropriate ceftazidime dosing regimens in neonates. A prospective PopPK study was conducted in neonates receiving ceftazidime. Blood samples were collected using the Capitainer quantitative DBS (qDBS) device, and concentrations were quantified by ultra-performance liquid chromatography–tandem mass spectrometry (UPLC–MS/MS). PopPK modeling was performed in NONMEM. Body weight (WT) and postmenstrual age (PMA) were incorporated a priori using allometric scaling and a sigmoidal maturation function. Additional covariates were evaluated using stepwise selection. Monte Carlo simulations were conducted to assess the probability of target attainment (PTA) for various dosing regimens against the target of 70% fT > MIC. A total of 140 qDBS samples from 72 neonates were analyzed. A one-compartment model adequately described ceftazidime PK. PMA significantly influenced clearance, while WT affected volume of distribution. Simulations indicated that, for neonates with PMA 32–42 weeks, regimens of 25 mg/kg every 6 or 8 h, and 75 mg/kg every 12 h achieved a PTA ≥90% for MIC ≤4 mg/L. A PopPK model for ceftazidime was successfully developed using qDBS samples from neonates. For neonates with PMA 32–42 weeks, dosing regimens of 25 mg/kg every 6 or 8 h and 75 mg/kg every 12 h are recommended to achieve optimal target attainment.

## INTRODUCTION

Neonatal sepsis remains a life-threatening condition and a leading cause of mortality and morbidity among newborns. The immaturity of the neonatal immune system and organ function significantly increases susceptibility to bacterial infections. Globally, neonatal infections account for approximately 46% of all under-five deaths and 49.2% of infection-related mortality (1). Preterm infants are at especially high risk of infection due to underdeveloped immunity and frequent exposure to nosocomial pathogens. Common gram-negative bacteria, including *Escherichia coli*, are the primary pathogens responsible for neonatal sepsis (2). Ceftazidime, a third-generation cephalosporin essential for treating neonatal gram-negative infections (3–6), displays time-dependent bactericidal activity. It demonstrates significant inhibitory effects against common pathogens, such as *Escherichia coli*. Its efficacy is determined by the percentage of time the unbound plasma concentration exceeds the MIC (%*f*T > MIC) (3, 7–12).

Ceftazidime is currently administered to neonates using a broad range of dosing regimens, with doses varying from 25 to 100 mg/kg and dosing intervals from every 6 to 12 h (6, 7, 11, 13). Neonatal pharmacokinetics are highly non-linear and strongly influenced by ongoing organ maturation and physiological immaturity (14, 15). Substantial interindividual variability—driven by differences in postmenstrual age, body weight, and organ function—further complicates dosing. Fixed dosing that does not account for these developmental and physiological factors may result in subtherapeutic exposure, treatment failure, emergence of antimicrobial resistance, or drug-related toxicity. Together, these considerations highlight the critical need for optimized, developmentally informed ceftazidime dosing strategies to ensure efficacy while minimizing risk in neonates.

Model-informed precision dosing supported by population pharmacokinetic (PopPK) modeling offers a rational approach to individualizing ceftazidime therapy by characterizing sources of pharmacokinetic variability. However, the ethical and practical constraints of blood sampling pose major challenges for conducting standard PK studies in neonates. Traditional protocols typically require 6–10 serial blood draws, which is problematic given neonates’ limited blood volume and the associated risk of iatrogenic anemia (16, 17). Venipuncture itself is technically difficult, often results in substantial and unpredictable blood loss (≥0.5 mL per procedure [18]), and may cause vascular injury and stress-related complications. Although several PopPK models for ceftazidime in neonates have been developed using conventional venipuncture sampling (11, 12), their clinical applicability remains limited.

To reduce the burden of blood sampling in infants, alternative strategies, such as opportunistic sampling and scavenged sampling, have been increasingly explored (19, 20). Opportunistic sampling collects research samples during clinically indicated blood draws and has been applied to ceftazidime PopPK modeling in neonates (7). However, it still relies on invasive venipuncture and typically yields sparse, irregularly timed samples that inadequately characterize key PK phases—particularly distribution and elimination—leading to potential bias in parameter estimation (19, 20). Scavenged sampling, which uses leftover blood from routine clinical tests, eliminates additional needle sticks but presents other limitations. Sampling times reflect clinical, not research, needs, resulting in suboptimal PK timepoints. Inconsistent sample handling or storage may cause drug degradation, and missing covariate data or inaccurate time documentation further compromise data quality (19, 20). Together, these issues limit the reliability and applicability of PopPK models developed solely through opportunistic or scavenged sampling (21, 22).

In this context, quantitative dried blood spot (qDBS) microsampling has emerged as a promising alternative to conventional venous sampling (7, 12). qDBS collects small, accurately metered blood volumes from heel or finger pricks onto specialized filter cards. This minimally invasive approach decreases procedure-related pain, reduces iatrogenic blood loss, and minimizes hematocrit-related bias (23). These advantages support standardized implementation across study sites and make qDBS particularly valuable for generating high-quality PopPK data in neonates.

This study aimed to evaluate the methodological applicability of qDBS for developing a population pharmacokinetic (PopPK) model of ceftazidime in Chinese neonates and to use the resulting model to optimize neonatal dosing strategies through Monte Carlo simulations.

## MATERIALS AND METHODS

### Study design and population

A prospective, single-center, open-label PopPK study in neonates was conducted at Xiamen Maternity and Child Health Care Hospital (from 2022 to 2024). Neonates with a postnatal age (PNA) ≤ 28 days who had confirmed or suspected bacterial infections requiring ceftazidime treatment, available intravenous access, and informed consent provided by legal guardians were eligible. Exclusion criteria included known hypersensitivity to ceftazidime or other cephalosporins, life-threatening infections, missing key baseline data, lack of parental consent, or any other condition deemed unsuitable by investigators. Demographic and clinical data, including body weight (WT), gestational age (GA), postnatal age (PNA), postmenstrual age (PMA), serum creatinine (SCR), and concomitant medications, were systematically collected.

### Sample collection and determination

Capillary blood sampling was performed from the heel using the Capitainer qDBS device (Capitainer AB, Sweden) at prescribed intervals (0–0.5, 2–3, and 4–6 h) after ceftazidime administration. A precise 10-μL aliquot was applied to the sampling card. All samples were air-dried, sealed, and maintained at −80°C until analysis. Ceftazidime concentrations were quantified using a validated ultraperformance liquid chromatography with photodiode array detection (UPLC-PDA) method (24). The assay was linear across the range of 0.25–50.0 μg/mL, with accuracy ranging from 92.5 to 101.4% and precision below 10.4%.

### PopPK modeling

A nonlinear mixed-effects modeling approach was implemented using NONMEM (v7.5, Icon Development Solutions, Ellicott City, MD, USA). Pre- and post-processing tasks were performed using PsN (v5.3.0, Uppsala University, Sweden) and Pirana (v2.9.9, Pirana Software & Consulting BV), and statistical analyses and visualizations were conducted in R (v4.4.1, R Foundation for Statistical Computing, Vienna, Austria). The first-order conditional estimation method with interaction (FOCE-I) was used for parameter estimation.

One- and two-compartment models were evaluated to describe the concentration-time data. The interindividual variability associated with PopPK parameters was characterized using an exponential model, which is assumed to follow a log-normal distribution with a mean of 0 and a variance of *ω*^2^. Residual variability was also modeled exponentially.

WT and PMA were incorporated a priori as covariates within the frameworks of the allometric and sigmoidal maturation models (25). Clearance (CL) and volume of distribution (V) were scaled allometrically based on WT, with exponents of 0.75 for CL and 1 for V, while PMA effects on CL were described using a sigmoidal maturation function (equations 1 and 2). For all other covariates, a stepwise approach was applied for statistical screening, with criteria set at *P* < 0.05 for forward inclusion and *P* < 0.01 for backward elimination.


(1) $$CL=TVCL\times {\left(\frac{WT}{70kg}\right)}^{0.75}\times \frac{PMA^{\text{Hill }}}{\left(PMA^{\text{Hill }}+TM_{50}^{\text{Hill }}\right)}\times e^{\eta_{CL\#}}$$

(2) $$V=TVV\times \left(\frac{WT}{70\;kg}\right)\times e^{\eta_{V}}\#$$

where TVCL represents the population typical value of clearance; TVV denotes the population typical value of volume of distribution; and *$e^{\eta_{CL}}$* and $e^{\eta_{V}}$ represent the interindividual variability of CL and V, respectively. TM50, representing the PMA corresponding to 50% maturation of clearance, was fixed at 47.7 weeks, while the Hill coefficient was fixed at 3.4.

### Model evaluation

Model was selected based on a comprehensive evaluation of objective function value (OFV), goodness-of-fit, and predictive performance. Goodness-of-fit was assessed through diagnostic plots, including observed versus population-predicted concentrations, observed versus individual-predicted concentrations, and conditional weighted residuals versus time or predictions. The predictive performance of the model was evaluated using visual predictive checks (VPC, *n* = 1,000 simulations). A non-parametric bootstrap (1,000 replicates) was performed to estimate the 95% confidence intervals of parameters and assess model robustness.

### Model-based simulation

To evaluate the probability of target attainment (PTA) of various ceftazidime dosing regimens in neonates, Monte Carlo simulations were performed based on the established PopPK model. The final PopPK model was re-implemented in R using the mrgsolve package (version 1.4.1). Twelve dosing regimens were assessed, comprising four dose levels (25, 50, 75, and 100 mg/kg) administered at three dosing intervals (every 6, 8, and 12 h) as 30-min intravenous infusions. These regimens were selected in accordance with published literature and standard neonatal dosing guidance, including NeoFax and the United States Pharmacopeia (3, 7, 11). A virtual neonatal cohort (*n* = 10,000 per subgroup) was generated using demographic data (PMA and WT) obtained from the neonatal database of Xiamen Maternity and Child Health Care Hospital. Simulations were conducted for each dosing regimen across three PMA subgroups: 32–35, 35–38, and 38–42 weeks. For comparison with other plasma-based PopPK models, unbound plasma concentrations—a necessary parameter—were derived from qDBS-based blood concentrations using equations 3 and 4, which define the blood-to-plasma ratio and the unbound plasma concentration, respectively. Specifically, equation 5 was obtained by substituting equation 3 into equation 4 to calculate the unbound plasma concentration (26):


(3) $$BP=\frac{C_{plasma}}{C_{blood}}\#$$

(4) $$C_{plasma,unbound}=f_{u}\times C_{plasma}\#$$

(5) $$C_{plasma,\;unbound}=f_{u}\times C_{blood}\times BP\#$$

where *C*_plasma, unbound_ is the unbound plasma concentration; *f*_*u*_ is the unbound fraction; *C*_plasma_ is the plasma concentration; *C*_blood_ is the blood concentration; and *BP* is the blood-to-plasma ratio of ceftazidime.

A pharmacokinetic/pharmacodynamics (PK/PD) target of 70% *f*T > MIC was employed for PTA evaluation of ceftazidime (7). MIC values ranging from 0.25 to 32 mg/L were assessed, covering susceptibility breakpoints relevant to ceftazidime (e.g., 4 mg/L for *Escherichia coli*) according to the European Committee on Antimicrobial Susceptibility Testing (EUCAST) database (https://mic.eucast.org/). A regimen was deemed clinically effective if it achieved a PTA ≥ 90%.

## RESULTS

### Study population

**Fig 1:**
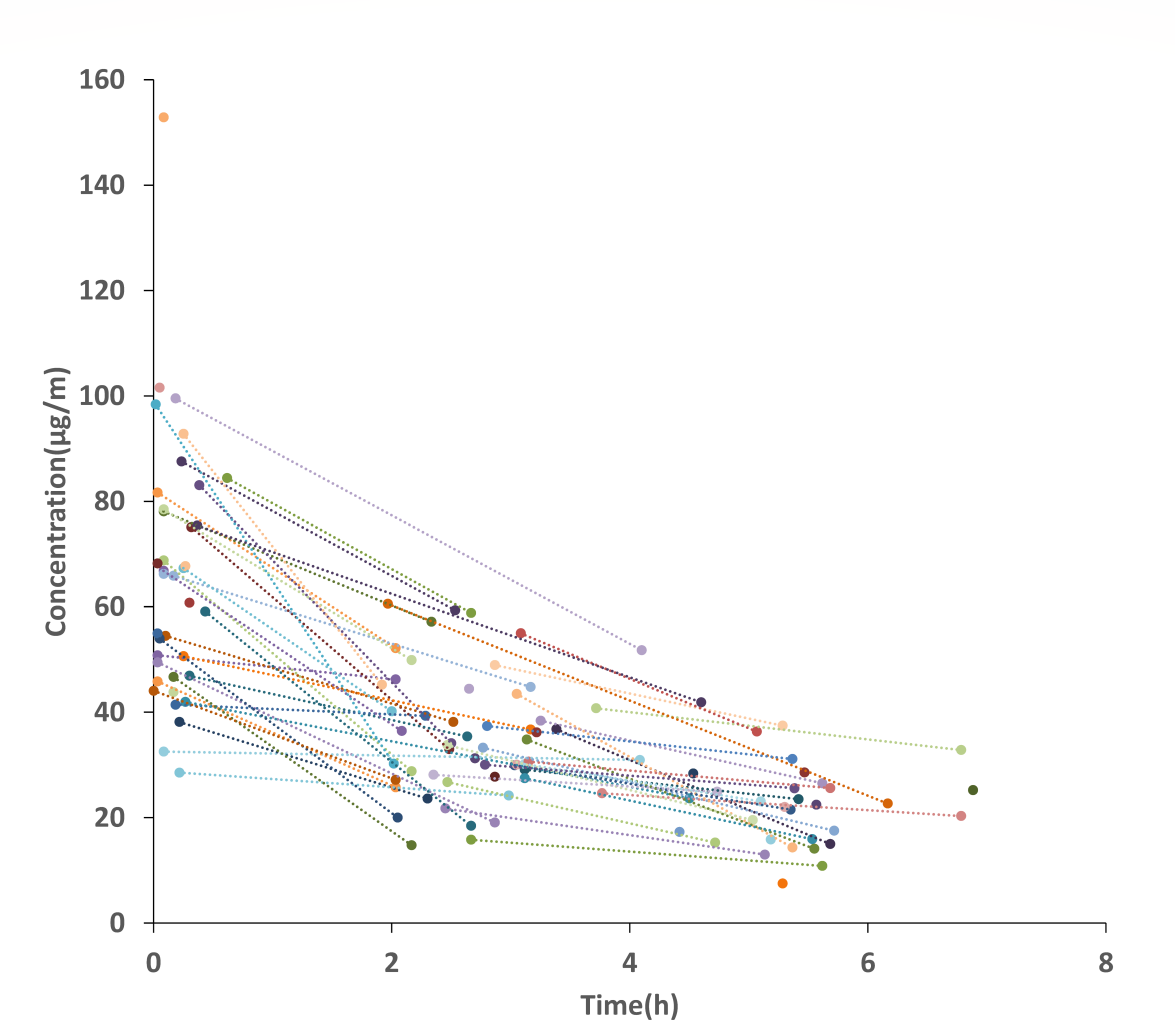
Scatter plot of ceftazidime concentration versus time after dose.

A total of 140 qDBS ceftazidime samples were collected from 72 neonates with informed consent. The median number of samples per patient was 2 (range: 1–4). Figure 1 depicts the ceftazidime concentration–time profile, and demographic and clinical characteristics are summarized in Table 1. The median PMA was 39.4 weeks (range: 32.6–41.3 weeks), and the median body weight was 3.2 kg (range: 1.8–4.2 kg).

**TABLE 1:** Demographics and clinical characteristics of the study neonatal patients

Characteristics	Median (range) or *n* (%)
Demographics and clinical information	
Gestational age (weeks)	39.4 (32.6–41.3)
Postmenstrual age (weeks)	39.7 (32.7–41.9)
Postnatal age (days)	1 (1–4)
Body weight (kg)	3.2 (1.8–4.2)
Serum creatinine (μmol/L)	56.7 (23.7–88.0)
Male/female	43 (59.7)/29 (40.3)
Preterm birth	14 (19.4)
Concomitant medications	
Meropenem	1 (1.4)
Vasoactive agents	13 (18.1)
Diuretics	3 (4.2)
Prenatal steroid exposure	4 (5.6)
Hydrocortisone	2 (2.8)
Ampicillin	13 (18.1)

### PopPK model building

The concentration–time data were best described by a one-compartment model. Only the a priori covariates PMA and WT were retained in the final model, as stepwise covariate selection did not identify any additional significant predictors. Incorporating PMA and WT improved the model fit (OFV of −153.8), reducing the OFV by 31.3 points compared with the base model.

The parameter estimates of the final population pharmacokinetic model showed acceptable precision, with all relative standard errors (RSEs) below 25% (Table 2). The bootstrap analysis converged in 967 of 1,000 runs (96.7%), and the median bootstrap estimates closely matched the final model estimates, all of which fell within the corresponding 95% confidence intervals—demonstrating good model robustness.

**TABLE 2:** Parameter estimates and bootstrap results for the final population pharmacokinetic model of ceftazidime^*b*^^,^^*c*^

Parameter	Final model	Bootstrap results	
	Estimate (RSE%) [shrinkage%]	Median	95% CI
Fixed effects			
CL (L/h/70 kg)	19.6 (4.2)	19.5	17.9–21.2
V (L/70 kg)	70.6 (7.5)	71.3	61.3–82.8
Interindividual variability			
CL (CV%)	28.1 (21.0) [23.3]	27.4	14.6–37.0
V (CV%)	42.4 (22.9) [33.9]	41.8	15.0–60.8
Residual variability			
Proportional error (%)^*a*^	22.3 (45) [31.6]	22.2	13.1–32.1

^*a*^ An exponential error model (additive error model in the log-transformed domain) was used to characterize the residual unexplained variability, which approximates to a proportional error in the normal domain.

^*b*^ CL: clearance, V: volume of distribution, and CI: confidence interval.

^*c*^ Coefficient of variation (CV%) was calculated as: $CV\%=\sqrt{e^{\omega^{2}}-1}\times 100\%$.

**Fig 2:**
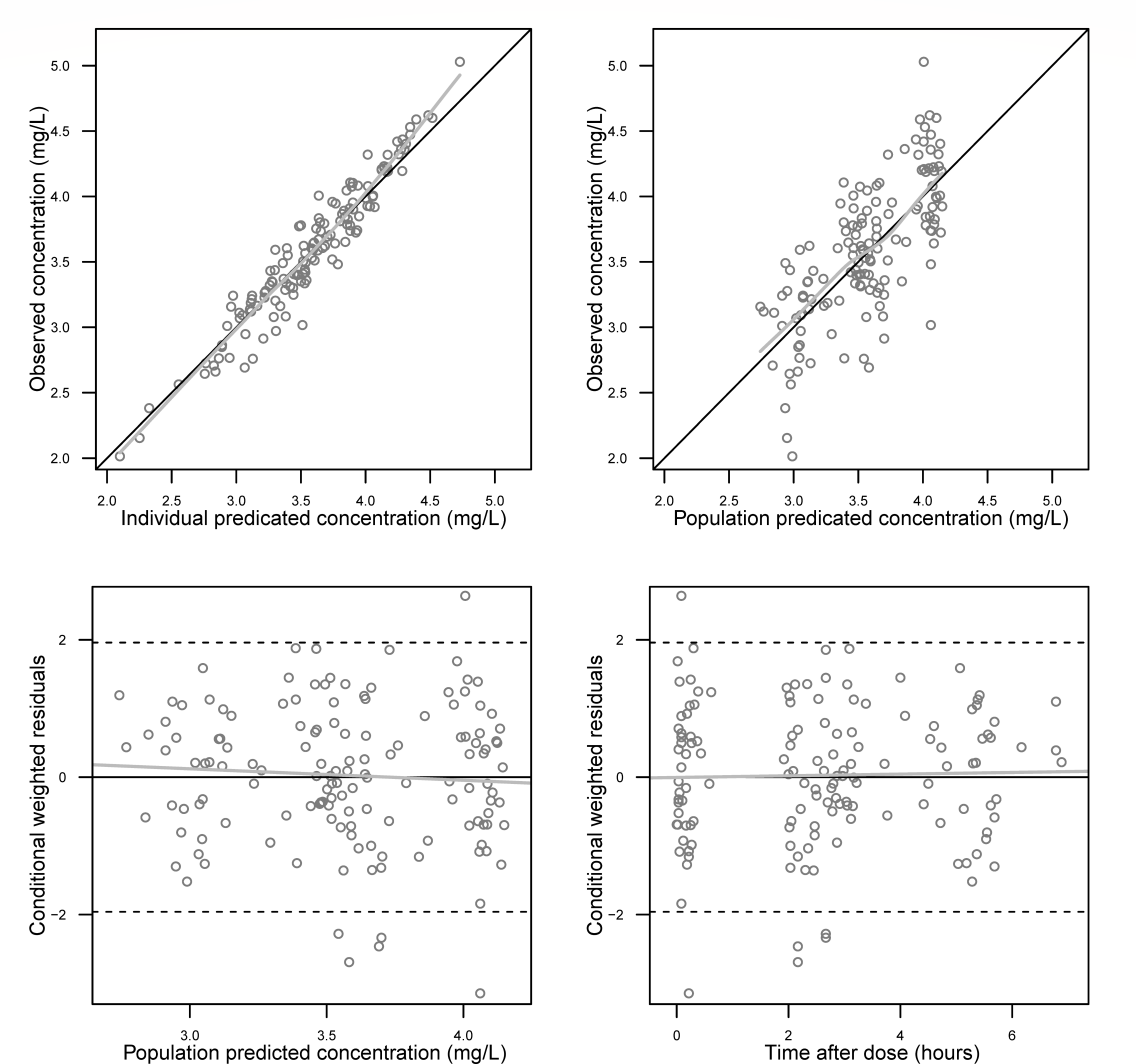
Goodness-of-fit plots of the final population pharmacokinetic model of ceftazidime. Top left panel: observed concentrations versus individually predicted concentrations. Top right panel: observed concentrations versus population-predicted concentrations. Bottom left panel: conditionally weighted residuals versus population predicted concentrations. Bottom right panel: conditionally weighted residuals versus time after dose. Solid gray lines represent locally weighted least-squares regressions.

**Fig 3:**
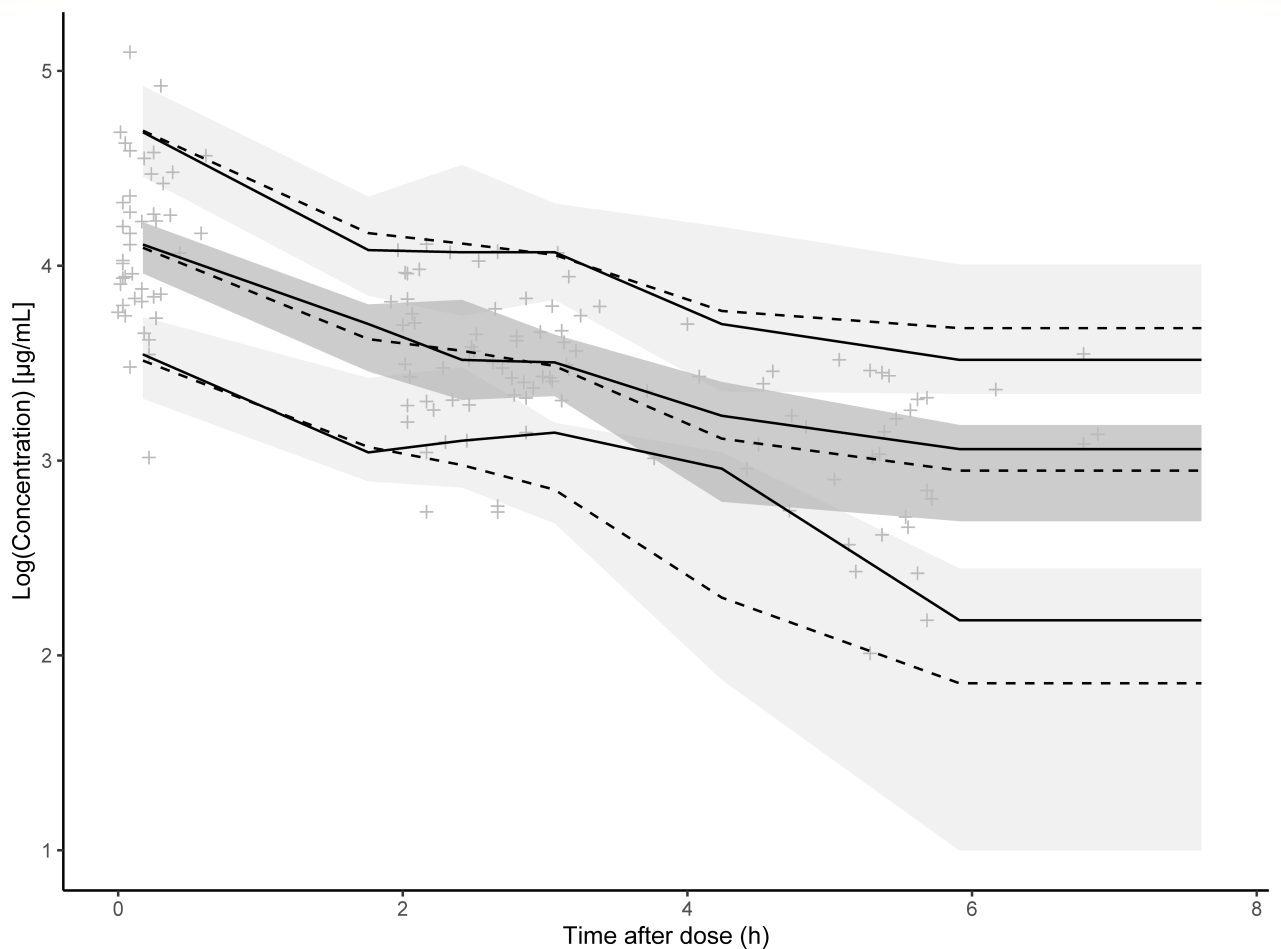
Visual predictive checks plot for the final population pharmacokinetic model of ceftazidime. The lower, middle, and upper solid lines are 5, 50, and 95% quantile lines of the observed concentrations, and the lower, middle, and upper dashed lines are the 5, 50, and 95% quantile lines of the predicted concentrations. The shaded areas represent the 95% confidence intervals for the prediction lines.

Goodness-of-fit plots (Fig. 2) showed that both population-predicted and individual-predicted concentrations were distributed closely along the line of identity, and the conditional weighted residuals were symmetrically distributed around zero without apparent systematic bias. The VPC (Fig. 3) further confirmed good agreement between the model predicted concentration-time curves and the observed data, with 94.3% of the observations falling within the 90% prediction interval, supporting the adequate predictive performance of the model.

### Model-based simulations

**Fig 4:**
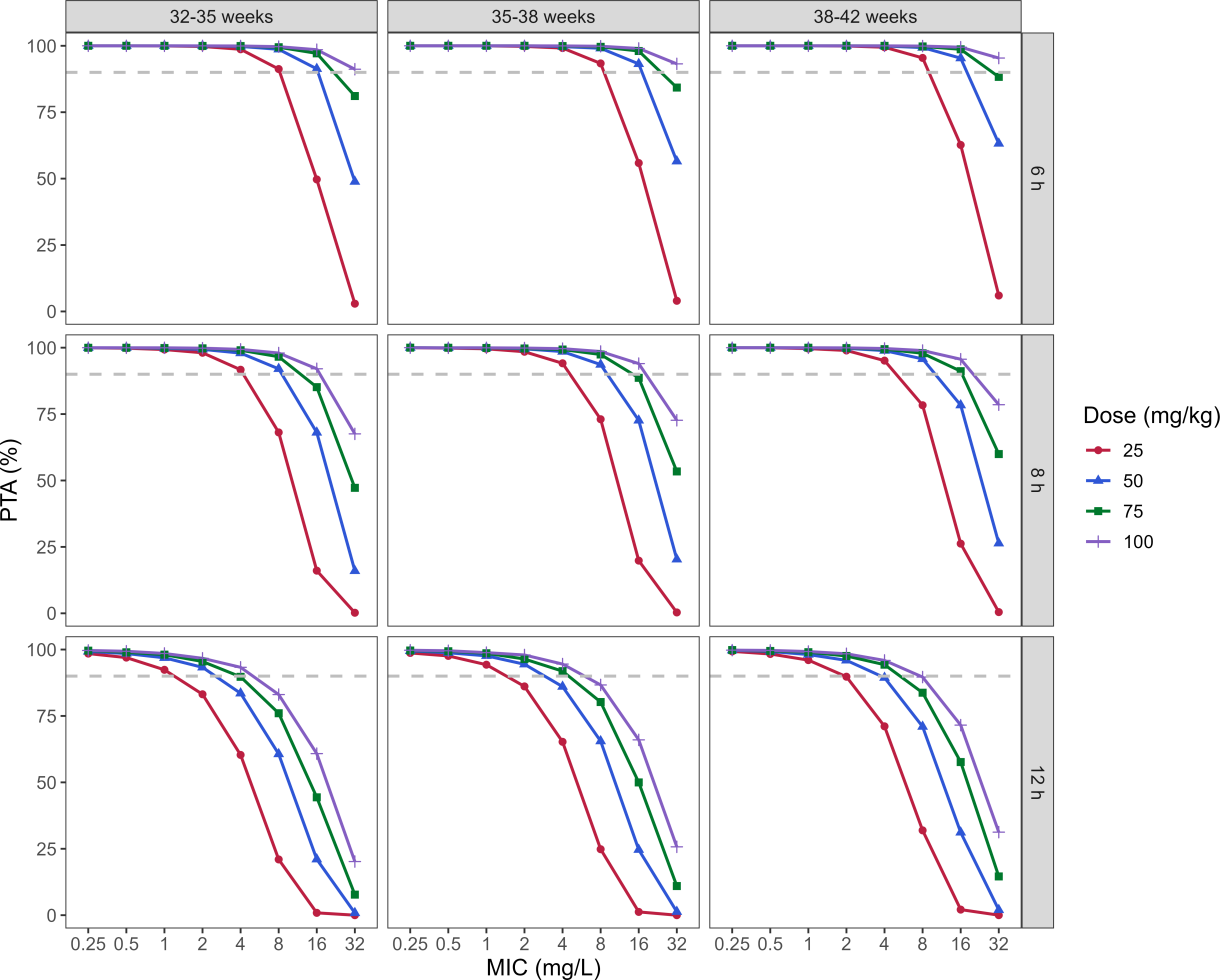
Probability of target attainment (PTA) for achieving 70% *f*T > MIC across MIC values for ceftazidime dosing regimens of 25, 50, 75, and 100 mg/kg administered as a 30-min intravenous infusion every 6, 8, or 12 h.

The PTA results for ceftazidime administered as a 30-min intravenous infusion are presented in Fig. 4. No meaningful differences in PTA were observed across PMA subgroups for any dosing regimen. For the PK/PD target of 70% *f*T > MIC at MIC values ≤4 mg/L, all regimens (25–100 mg/kg) achieved PTA ≥90% at 6- and 8-h dosing intervals, whereas only the 75- and 100-mg/kg regimens met this target when the interval was extended to 12 h.

## DISCUSSION

The qDBS microsampling technique was adopted to address the limitations of traditional venipuncture-based sampling in pediatric pharmacokinetic studies. Whole-blood concentrations obtained from qDBS samples were used directly to develop the ceftazidime PopPK model, while unbound plasma concentrations were derived during simulations using the blood-to-plasma concentration ratio and the plasma unbound fraction. Alternatively, qDBS-derived blood concentrations can be converted to plasma concentrations prior to modeling. After applying the blood-to-plasma ratio for parameter conversion, the fixed-effect estimates and variability components were comparable to those from the blood-based model (data not shown), consistent with findings from our previous neonatal ampicillin pharmacokinetic study (23).

Covariate screening was performed using a physiology-informed strategy. During this process, SCR was identified as the only continuous covariate with missing data, which was observed in 2 of 72 neonates (2.8% of the study cohort). Missing values were imputed with the overall mean from the remaining 70 neonates—an approach widely accepted for PopPK modeling with low missing rates (≤5%). Body weight effects on CL and V were incorporated using allometric scaling, and the developmental influence of PMA on CL was modeled using a sigmoidal maturation function (27, 28). Because ceftazidime is primarily eliminated by the kidneys, the maturation model was adapted from the glomerular filtration rate model proposed by Rhodin et al. (25), with TM₅₀ (47.7 weeks) and the Hill coefficient (3.4) fixed to literature values, as our data set did not support reliable estimation of these parameters. This physiology-based approach adequately described ceftazidime pharmacokinetics in Chinese neonates and demonstrated good predictive performance. For comparison, we also evaluated a data-driven stepwise covariate selection process without incorporating any a priori covariates. This approach identified body weight as the only significant covariate on CL and yielded a model with an OFV of –143.9. However, this OFV was 9.9 points higher than that of the physiology-informed final model, and the stepwise model was, therefore, not selected. Findings about covariates from previous studies have varied. Wang et al. used a stepwise approach without prior covariates and identified PMA and WT as significant predictors (12), whereas Li et al. incorporated WT a priori *via* allometric scaling and subsequently identified GA and PNA as additional covariates (7), and van der Veer et al. included neonatal body temperature and PNA as covariates for CL (11). The variability in identified covariates across studies likely reflects differences in patient characteristics and study populations.

In the final PopPK model, the typical CL and V, based on qDBS concentrations, were estimated as 19.6 L/h/70 kg and 70.6 L/70 kg, respectively. To enable comparison with plasma-based PopPK studies, parameters were converted using a reported blood-to-plasma concentration ratio of 0.72 for ceftazidime (26). After standardizing covariates to median values (PMA = 39.4 weeks, PNA = 1 day, and WT = 3.2 kg), substantial interstudy variability in plasma clearance estimates was observed. The plasma CL predicted by our model (0.49 L/h/kg) was higher than those reported by Li et al. (0.224 L/h/kg) (7) and van der Veer et al. (0.144 L/h/kg) (11), but comparable to the estimate from Wang et al. (0.301 L/h/kg) (12), which incorporated similar covariates (PMA and WT). In contrast, volume of distribution estimates showed no major discrepancies across studies (current study: 2.32 L/kg; Li et al.: 1.41 L/kg; Wang et al.: 2.13 L/kg; van der Veer et al.: 1.65 L/kg) (7, 11, 12).

Our simulations showed that three dosing regimens—25 mg/kg every 6 h, 25 mg/kg every 8 h, and 75 mg/kg every 12 h—achieved the predefined pharmacodynamic target without excessive exposure, providing adequate antibacterial coverage for pathogens with MIC ≤4 mg/L, such as *Escherichia coli*. In contrast, the United States Pharmacopeia-recommended regimen of 30 mg/kg every 12 h did not meet the PK/PD target in our study cohort. For pathogens with higher MICs (≤8 mg/L, e.g., *Pseudomonas aeruginosa*), two optimized regimens (25 mg/kg q6h or 50 mg/kg q8h) were identified in our simulation to achieve the target PTA. This dosing strategy was consistent with the work of Shi et al. (3), who reported that a 50 mg/kg q8h regimen is required for an MIC of 8 mg/L. In contrast, the regimens proposed by van der Veer et al. (11) (25 mg/kg q24h or 10–15 mg/kg q12h) failed to achieve the PK/PD target in our study cohort. Substantial heterogeneity exists among published ceftazidime dosing recommendations for neonates, largely reflecting differences in estimated PopPK parameters and patient stratification strategies. For example, Li et al. stratified neonates by postnatal age (PNA ≤3 days versus >3 days) and gestational age (GA ≤37 weeks versus >37 weeks) (7). Wang et al. used a combined stratification based on body weight (<2.5, 2.5–3.5, and >3.5 kg) and postmenstrual age (<250, 250–300, and >300 days) (12). In contrast, van der Veer et al. stratified patients by body temperature status (hypothermia, rewarming, normothermia) (11). Notably, optimal ceftazidime dosing in neonates aged 32–42 weeks depends not only on total daily dose but also on renal function (which governs clearance) and fluid status (which influences volume of distribution). Reduced renal clearance may lead to drug accumulation, favoring dose reduction or prolonged dosing intervals, whereas fluid overload associated with an increased volume of distribution may lower trough concentrations, potentially compromising %fT > MIC and necessitating higher loading or maintenance doses. In addition, dosing frequency substantially affects peak and trough exposure; regimens with similar daily doses but different intervals (e.g., 25 mg/kg every 6–8 h versus 75 mg/kg every 12 h) may yield markedly different peak concentrations and PTA profiles. Therefore, the dosing regimens evaluated in this study should be interpreted as population-based reference scenarios, and individualized dosing guided by renal function, fluid status, and therapeutic targets is recommended.

We acknowledge some limitations. First, we note that the lack of microbiological outcomes (e.g., pathogen identification and MIC data) and clinical endpoints (e.g., clinical response and safety outcomes) limited comprehensive PK-PD integration and precluded direct assessment of exposure-response and exposure-toxicity relationships in this neonatal population. Second, the optimized ceftazidime dosing regimens proposed herein are restricted to the management of systemic gram-negative sepsis in neonates and fail to account for cerebrospinal fluid (CSF) penetration—a critical pharmacokinetic property for the treatment of meningitis and other central nervous system (CNS) infections. Given that CSF penetration may occur in neonates with sepsis, careful consideration is warranted when extrapolating these regimens to neonates with suspected or confirmed CNS involvement. Third, external validation was not performed in this study, which is often difficult to achieve in neonatal pharmacokinetic studies due to ethical and practical constraints. Therefore, the simulation and probability of target attainment results should be interpreted as supportive and exploratory, and future studies with independent data sets are warranted to further assess the model’s external predictability.

### Conclusion

This study developed a PopPK model for ceftazidime in Chinese neonates using qDBS micro-sampling. The model incorporated postmenstrual age and body weight as a priori covariates to account for developmental changes in pharmacokinetics. For neonates with a postmenstrual age of 32–42 weeks, our findings suggest that dosing regimens of 25 mg/kg every 6 or 8 h or 75 mg/kg every 12 h are appropriate options.
